# Rosenkrans’ Case of Old Dislocation

**Published:** 1848

**Authors:** H. Rosenkrans

**Affiliations:** Ottawa, Illinois


### ARTICLE II.

*Old Dislocation of the Humerus Mistaken for recent Injury, and consequent Failure of the Attempt to reduce.* By H. Rosenkrans, M.D., of Ottawa, Illinois.

On the evening of the 28th ult., I was called to see Mr. R. A., a man of middle age, stout and muscular, Upon my arrival, I found him complaining of slight pain and soreness of the arm midway between the elbow and shoulder; he had also a bruised eye. The injury had been received in jumping from a wagon, and falling partly on the left arm. The arm was found extended at right angles with the body. He did not complain of pain in the shoulder, or of the injury affecting it; but, upon examination, I found that the shoulder was dislocated, the head of the bone resting below and anterior to the glenoid cavity, and under the pectoralis major muscle. I was surprised to find with what freedom I could move the arm; the patient complaining only when I happened to touch the lower third of the humerus, the part recently injured by the fall. Being satisfied of the correctness of my diagnosis, I commenced attempting its reduction. I first desired to bleed the patient; to this he objected, if it could be reduced without, consequently, I did not insist upon the bleeding, remarking to him, however, that it would require more power to overcome the resistance of the muscles, to which he seemed willing to submit, rather than to the venesection. I then had him placed on the floor, and, seating myself at his left side, placed my heel in his axilla and seizing his left elbow made what traction I could for a few minutes but to no effect. I then had him extended on a bench, with an extending bandage applied above the elbow and a sheet, passed under the axilla of the affected side and over the opposite shoulder, having care to prevent it from pressing against the pectoral and latissimue dorsi muscles so as not to render useless the force applied at the elbow. All things in readiness, a man placed himself at the end of the sheet so braced against a door as to counteract the force of three men at the elbow. This amount of force was applied and kept up for some minutes, the patient complaining all the while. This did not produce the least movement of the head of the bone. I then ordered them to desist. We now sent for Dr. Howland. The patient having been bled to the amount of thirty ounces, the Doctor then placing his foot in the axilla, made what extension he could by taking hold of the patient's elbow, rotating the arm. Whilst manipulating in this way, the Dr. thought he heard the bone slide into place; the patient remarked, however, that it was his "elbow that snapped." I could not perceive that there was any alteration in the position of the head of the bone. I at this time began to conclude that the dislocation might be an old one, and called the attention of Dr. Howland to the atrophied condition of the deltoid muscles. I then enquired of the patient if he had ever noticed a difference in the two shoulders, to which he replied that he had for years past, and he then also stated that he had received an injury of that shoulder some fifteen years since, and that ever since that time he had had but imperfect use of it, not being able to raise weights, or hardly to bring the arm into a horizontal position. We left the patient, satisfied that the dislocation had existed from the time of the former injury.

Now the question arises—was there too great a tardiness in discovering it to be an ancient dislocation, under the circumstances? We certainly should not have attempted its reduction if we had known its antiquity, unless for consecutive dislocation, which, I think, did not obtain. The patient complained, for a few days, of pain and soreness in the elbow, bnt the shoulder did not trouble him, if we except the slight stiffness and soreness in the muscles, occasioned by the pulling to reduce the dislocation.

